# Neonatal meconium aspiration syndrome with transient Hyperreactive airways and socioeconomic challenges: a case report

**DOI:** 10.1093/omcr/omaf067

**Published:** 2025-06-27

**Authors:** Carlos Diaz Q, Marcos Orellana, Andrea Argueta, Pedro Chajon

**Affiliations:** Research Medical Department, Universidad Francisco Marroquin, Guatemala City 01011 Guatemala; Research Medical Department, Universidad Francisco Marroquin, Guatemala City 01011 Guatemala; Research Medical Department, Universidad Francisco Marroquin, Guatemala City 01011 Guatemala; Research Medical Department, Universidad Francisco Marroquin, Guatemala City 01011 Guatemala

**Keywords:** meconium aspiration syndrome, Hyperreactive airways, neonatal respiratory distress, resource-limited care

## Abstract

This report presents a unique case of Meconium Aspiration Syndrome (MAS) in a neonate who developed transient hyperreactive airways syndrome (THAS), a rare complication marked by episodic bronchospasm and wheezing after MAS resolution. The patient, initially born in a private hospital in Guatemala and transferred to a community hospital due to financial constraints, improved with CPAP therapy but required bronchodilators for intermittent respiratory exacerbations. This case highlights the diverse manifestations of MAS and the need for careful monitoring of potential complications, particularly in resource-limited settings.

## Introduction

Meconium aspiration syndrome (MAS) is a serious condition that occurs when a newborn inhales meconium into the lungs during or shortly after birth, which can cause respiratory distress and various complications. It typically occurs when the infant passes meconium into the amniotic fluid before or during labor, often in the presence of fetal distress. The inhaled meconium obstructs the airways, leading to inflammation, chemical pneumonitis, and in severe cases, pulmonary hypertension. MAS is associated with high morbidity, particularly when complicated by infections or persistent pulmonary hypertension of the newborn (PPHN).

MAS affects 2%–10% of neonates exposed to meconium-stained amniotic fluid, posing a risk for severe respiratory complications, including chemical pneumonitis, pulmonary hypertension, and air leaks. [1] While these are well-documented, transient hyperreactive airways syndrome (THAS) is an underreported sequela of MAS. THAS, characterized by episodic bronchospasm and wheezing, is hypothesized to result from airway hyperresponsiveness secondary to meconium-induced inflammation.

This case report describes a neonate with MAS complicated by THAS, managed successfully in a resource-limited environment, providing insights into clinical adaptability in low-resource settings.

## Case presentation

**Figure 1:**
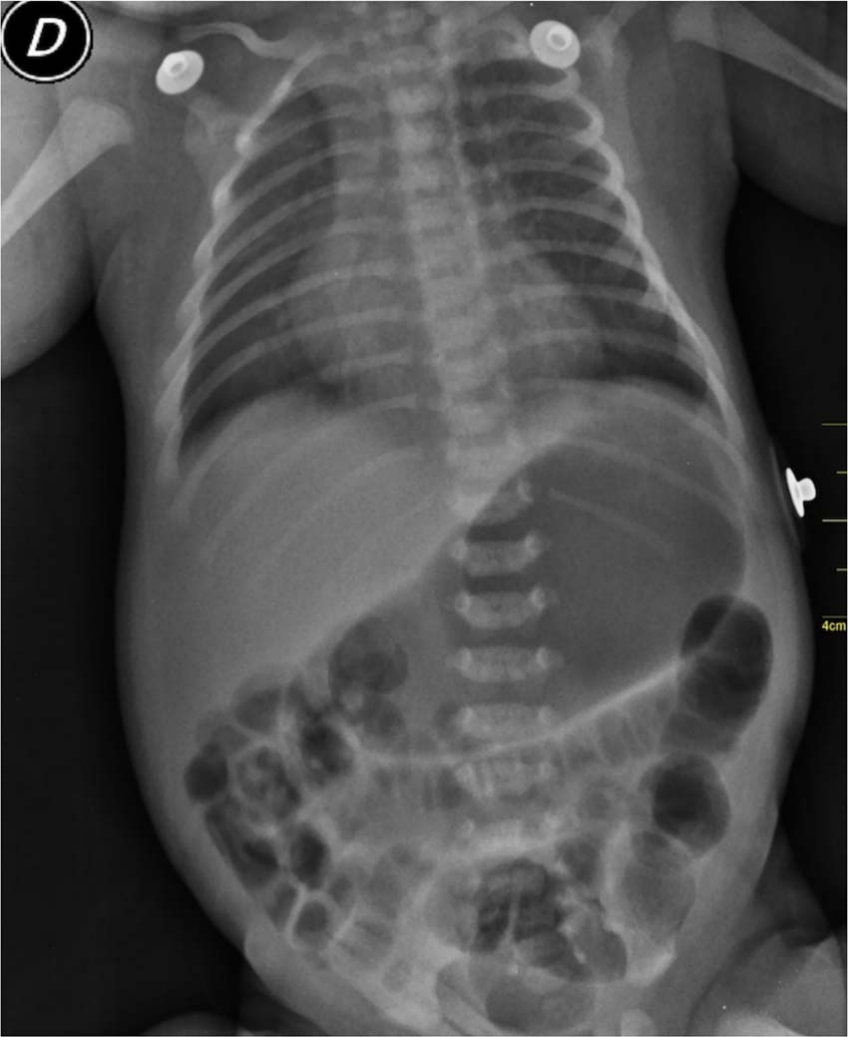
Chest X-ray of the neonate showing diffuse patchy infiltrates consistent with meconium aspiration syndrome (MAS), indicative of widespread pulmonary involvement. Dilated bowel loops on the CXR are also observed, suggesting underlying gastrointestinal dysfunction, which may have contributed to the clinical presentation.

A 2-week-old male neonate from a rural, low-income area in Guatemala was referred to a community hospital with MAS. X-ray revealed diffuse patchy infiltrates consistent with MAS and mild bowel distension (Fig. 1), likely due to transient intestinal dysmotility rather than gastrointestinal dysfunction. This finding was essential for diagnosis and management.

The intrapartum course was notable for a 38-week gestation pregnancy complicated by oligohydramnios and fetal distress, leading to an emergency cesarean section. Thick meconium-stained amniotic fluid was observed upon rupture of membranes. The neonate was delivered limp, with poor respiratory effort, requiring immediate resuscitative measures. Initial steps included endotracheal suctioning before spontaneous respirations were established, followed by positive-pressure ventilation for persistent bradycardia. The APGAR scores were 3 at 1 min and 6 at 5 min. Due to continued respiratory distress, the neonate was stabilized on supplemental oxygen before being transferred to a higher-level facility for advanced respiratory support.

Echocardiography was performed and revealed mild pulmonary hypertension (estimated pulmonary artery pressure of 40 mmHg) with normal cardiac anatomy and function. There was no evidence of structural congenital heart disease, intracardiac shunts, or significant left-to-right flow. The right ventricle appeared mildly hypertrophied but with preserved systolic function. Given the absence of persistent hypoxemia, differential cyanosis, or hemodynamic instability, no further cardiac intervention was required, and the pulmonary hypertension resolved as the neonate’s respiratory status improved.

On admission, the neonate exhibited severe respiratory distress with tachypnea, nasal flaring, and cyanosis. Continuous Positive Airway Pressure (CPAP) therapy with an FiO₂ of 60% was initiated. Laboratory results revealed mild metabolic acidosis (pH 7.28, HCO₃^−^ 17 mmol/l) and elevated inflammatory markers. Initial chest X-rays showed diffuse patchy infiltrates consistent with MAS.

The infant responded rapidly to CPAP therapy, with significant clinical improvement noted by day 3, enabling weaning to room air by day 5. However, intermittent episodes of bronchospasm and wheezing were observed during the recovery phase. These episodes, characterized by sudden-onset respiratory distress with wheezing, were relieved with inhaled bronchodilators (albuterol) and supplemental oxygen. No infectious etiology was identified, and echocardiography, though unavailable, was deemed unnecessary due to stable oxygen saturation and absence of clinical signs of pulmonary hypertension.

The patient is suspected to have meconium ileus because dilated bowel loops with minimal hydro-aerial levels can be seen on x-ray. The ‘soap bubble’ pattern, caused by mixing of trapped air with the dense meconium in the bowel loops, can be seen. Thick meconium also leads to this diagnosis.

The family’s financial situation necessitated transfer to a community hospital for continued care. Despite limited resources, the neonate thrived, and the hyperreactive airway episodes resolved spontaneously within two weeks without further intervention.

## Discussion

### THAS in the context of MAS

Although airway hyperresponsiveness is well-documented in older children and adults, its occurrence in neonates recovering from MAS is rarely described. The exact mechanism remains unclear but may involve residual airway inflammation, disruption of epithelial integrity by aspirated meconium, and altered vagal tone. The self-limiting nature of THAS in this case aligns with a hypothesis of temporary airway remodeling, with inflammation subsiding as the lungs recover. [2, 3]

### Implications for clinical management

The intermittent bronchospasm observed in this neonate highlights the need for heightened vigilance during the recovery phase of MAS. Early recognition and prompt management with bronchodilators ensured rapid symptom resolution without progression to more severe respiratory compromise. [4] This case underscores the importance of individualized care, particularly in resource-limited settings where advanced diagnostic tools may not be available. [5]

The transfer from a private to a community hospital highlights the impact of socioeconomic factors on neonatal care in Guatemala. Despite limited resources, effective use of available tools ensured a positive outcome. Echocardiography is crucial for diagnosing MAS complications like PPHN, distinguishing it from congenital heart disease. While the community hospital could confirm the diagnosis, treatment was limited due to the lack of CPAP and essential medications, underscoring the challenges of resource-limited settings.

### Management of MAS

The management of MAS often includes supportive interventions such as Continuous Positive Airway Pressure (CPAP) to improve oxygenation and ventilation. This neonate’s rapid response to CPAP therapy was consistent with the typical management approach for MAS. However, the development of intermittent bronchospasm and wheezing during the recovery phase required additional attention. Early recognition of these episodes and prompt treatment with inhaled bronchodilators (albuterol) allowed for the rapid resolution of symptoms, highlighting the importance of timely and appropriate intervention in neonates with MAS. [5]

Although the majority of neonates recover uneventfully, complications such as THAS can occur and should be addressed promptly to prevent further respiratory distress. [6] The use of bronchodilators for symptomatic management of THAS is a reasonable and effective approach, as seen in this case. Moreover, monitoring for signs of pulmonary hypertension or persistent air leaks is essential in the management of MAS, though they were not observed in this neonate. [7, 8]

Echocardiography plays a crucial role in the evaluation of neonates with MAS, as it helps identify persistent pulmonary hypertension of the newborn (PPHN), a major complication that can contribute to respiratory distress and wheezing. [9] In this case, the patient had mild pulmonary hypertension on initial echocardiography, which resolved alongside the improvement of respiratory symptoms. The transient wheezing episodes observed during recovery may have been exacerbated by residual pulmonary hypertension and airway inflammation, explaining the positive response to inhaled bronchodilators. While steroids are not routinely used in MAS, their role in modulating airway inflammation and hyperresponsiveness could be considered in select cases, particularly those with persistent wheezing despite resolution of the acute phase.

## Conclusion

This report describes a rare complication of MAS: transient hyperreactive airways syndrome (THAS). The neonate’s accelerated recovery with CPAP, coupled with episodic bronchospasm during convalescence, highlights the diverse spectrum of MAS and the critical role of vigilant post-acute care. The case underscores the importance of tailoring interventions to resource-limited settings and raises awareness of THAS as a potential sequela of MAS.

## Data Availability

All data supporting this case report are included in the article, with no additional data available due to patient confidentiality.
